# Interdisciplinary Management of Teeth With a Complicated Crown Fracture: A Case Report With Follow-Up Checklist

**DOI:** 10.7759/cureus.32889

**Published:** 2022-12-23

**Authors:** Srikurmam Manisha Sai Kiran, Soumya S Shetty, Sanjyot Mulay, Riya A Parwani, Komal Gupta

**Affiliations:** 1 Department of Conservative Dentistry and Endodontics, Dr. D.Y.Patil Dental College and Hospital, Dr. D.Y.Patil Vidyapeeth, Pune, IND

**Keywords:** intraradicular splint, gingival recession, dental trauma, crown fracture, biomimetic dentistry

## Abstract

Traumatic injuries resulting in damage to the teeth and associated structures have a grave psychological impact on the patient and hence, these require timely intervention. The fracture extent determines the appropriate treatment plan for the patient. Crown fractures can be treated aesthetically without the invasion of the biological width with meticulous interdisciplinary management. The objective of this article is to report a case of a 27-year-old Indian male patient who presented to our hospital the following day, post-trauma to the anterior teeth. On clinical examination, two teeth were found to have undergone fracture that involved the pulp chamber. Considering the overall oral health, pulpal and periodontal health, availability of the fragments, and invasion of the biological width, a tailor-made treatment plan was devised. The fractured crown fragments were reattached surgically, using a glass fiber post that resulted in an aesthetic biological restoration. This treatment has been successfully managed by an interdisciplinary approach. At the 18th-month follow-up, clinical and radiological examinations suggest a successful outcome.

## Introduction

Traumatic injuries resulting in damage to the teeth and associated structures have a grave psychological impact on the patient and hence require timely intervention. It is a common consequence of any trauma directed to the anterior region of the face wherein the teeth may take up most of the forces and fracture [1]. The literature suggests that the most susceptible tooth to traumatic injury is the maxillary central incisor with a prevalence being 37%, which sustains approximately 80% of dental injuries followed by the maxillary lateral and the mandibular central and lateral incisors [2,3]. The injury to the coronal portion results in either complicated or uncomplicated fractures that may involve the enamel, dentin, pulp, and periodontal tissues, and hence there must be modifications of the treatment strategies to allow for multi-disciplinary management [1]. The extent of the fracture dictates the management of the injury to the tooth. Treatment ranges from a simple composite adhesive restoration to reattachment of the remaining tooth portion and coronal fragment using a fiber post, considering the fragment has enough tooth structure and is not grossly destructed [4].

This article aims to report a well-documented case of a complex crown fracture in which there was a timely intervention, where the fractured crown of the tooth itself has been used as a restoration. There is sufficient evidence to support the devised treatment plan in which the tooth has been used as a biological restoration, which can be a long-term restorative option [5]. In the current case, a 27-year-old male patient had a fall one day before reporting to the hospital that resulted in a fracture of his two upper anterior teeth. The fracture was managed esthetically with minimal biologic costs. This report describes a systematic approach to diagnosing, devising appropriate treatment plans, and step-by-step management of a complex case involving more than one tooth at a time. The highlight of this report is the successful management of the post-operative gingival recession that had arisen during the follow-up period.

This case report was prepared according to the PRICE 2020 Guidelines [6].

## Case presentation

History

A 27-year-old male patient has reported to our hospital with a chief complaint of fractured teeth in the upper anterior region after having met with an accidental fall approximately 18 hours back. He had continuous and unbearable pain with no missing teeth. The patient had not taken any medication. Past medical and dental history was irrelevant. The patient’s primary concern was the pain and he wished to treat the teeth as aesthetically pleasing as possible.

Diagnostic procedures

**Figure 1:**
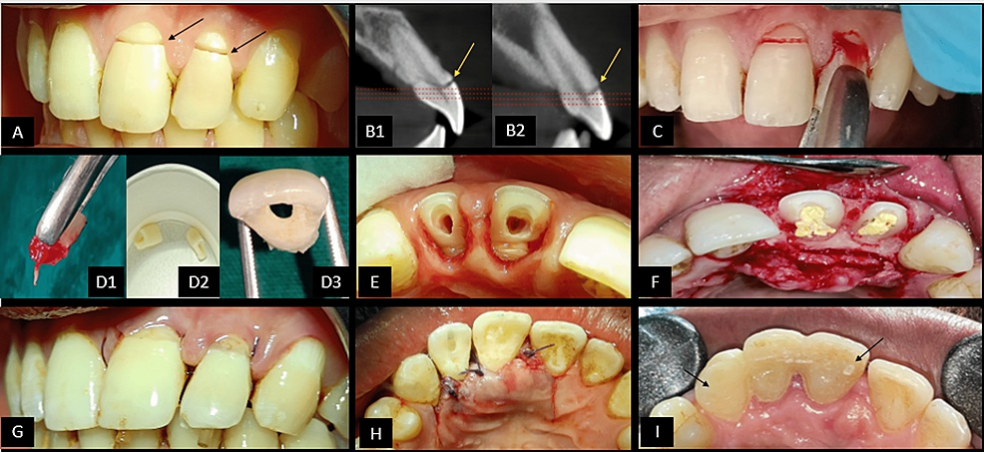
Pre-operative images, surgical intervention steps and post-surgical steps. (A) Arrows pointing at the horizontal fracture line with 21-22. (B1, B2) Pre-operative CBCT images in sagittal section showing oblique fracture line extending palate-cervically. It can be seen that the fracture line is extending supra-crestally and there is no concomitant root fracture. (C) Extraction of fragments using forceps. (D1-D3) Extracted fragment with removal of pulp in-toto; extracted fragments stored in saline; Preparation of a vent hole in fragment. (E) Palatal view after extraction of the fragments. Note the fracture site of the fragments seen horizontally on crown of each tooth. (F) Muco-periosteal flap raised from 11 to 22. Note that the root canal opening is closed and isolated using Teflon tape to prevent contamination of the root canals. Also note the controlled bleeding seen at the surgical site that has been achieved using gel foam. (G) Vertical mattress sutures placed using 3-0 vicryl sutures seen in labial view. (H) Vertical mattress sutures placed using 3-0 vicryl sutures seen in labial view. (I) Ribbond splint extending palatally from 21 to 23 to stabilize the teeth and prevent drifting away from each other.

On clinical examination, a fracture line was visible on the labial surfaces of two teeth i.e., the Maxillary left central incisor (21) and maxillary left lateral incisor (22) (Figure 1A). The Crown portion of the teeth was seen separated from the rest of the tooth structure, just attached to the marginal gingiva. The oblique fracture line was extending sub-gingivally and supra-crestally with no associated bone or root fractures. There was the absence of pain on palpation, mobility of teeth, swelling, or any pus discharge. The patient was advised a Cone beam computed tomography (CBCT) scan to understand the Bucco-palatal extent of the fracture line from a 3-dimensional perspective. Pre-operative radiographs and clinical images were taken to record the case and discuss treatment options with the patient. In the CBCT scan (iCAT 17-19, 8.9s, 120kV, 5mA), it was noted that the fracture line was passing through the pulp chamber reaching the palatal cervical region in Bucco-palatal direction of 21 and 22 (Figures 1B1, 1B2). There is the absence of concomitant transverse root fracture in both teeth. The CBCT has been an invaluable diagnostic imaging aid that has been beneficial in the current case to visualize the fracture, determine the prognosis of the treatment, plan the treatment and accurately measure the remaining length of the root portion of the teeth. From the above findings, 21 and 22 were diagnosed to be having complicated crown fractures [7] or Ellis Class III fractures [7].

The treatment options that were identified [8] are re-attachment of the crown fragments using a fiber post and composite build-up, re-attachment of the crown fragments using a fiber post and crown prosthesis, orthodontic extrusion followed by post and core with a final crown prosthesis, surgical extrusion followed by post and core with a final crown prosthesis, extraction followed by fixed prosthesis, extraction followed by removable prosthesis or extraction followed by an implant.

After discussing with the patient regarding the clinical scenario, financial aspects, a number of visits to the dental and the patient had reported at the earliest, we had the option to do single-visit endodontics and re-bond the crown fragment, thus salvaging the natural tooth for as long as it can be retained without causing any functional or aesthetic harm. The patient’s informed consent was then taken after explaining the procedure and prognosis. After evaluating the extent of the fracture line, endodontic-periodontic status of teeth, invasion of biological width, the fit of the fragments when approximated during the examination, stage of root formation at the time of fracture, concurrent hard or soft tissue injuries, the prognosis was considered as “fair” [9].

Clinical steps

Phase-wise treatment was carried out in a systematic manner. In phase one, the endodontic procedure was conducted and in phase two, the surgical procedure was conducted in which the crown fragment reattachment was done.

Phase 1 (Single Visit Endodontics)

Local anesthesia was administered (infraorbital nerve block and palatal infiltration) using 2% lignocaine hydrochloride (1:200,000 adrenaline) (Xicaine, ICPA Health, Gujarat, India). Fractured crown fragments were extracted using forceps and a moon probe to separate the gingival fibers facilitating the separation of a fragment from the rest of the tooth (Figures 1C, 1D1). Hemostasis was achieved by applying a hemostat (Viscostat, Ultradent). The extracted fragments were then cleaned and stored in 0.9% Normal saline (0.9% NaCl) to prevent dehydration (Figure 1D2). Root canal treatment was started by noting the initial working length using an electronic apex locator (Root ZX mini, J Morita) with #10-K stainless steel file (MANI), which was confirmed radiographically (working length of 21 is 18 mm and 22 is 17mm). The glide path was achieved using a 10-K stainless steel file followed by the cleaning and the shaping procedure was performed with #30 4% Ni-Ti rotary file for tooth 21 and #25 4% Ni-Ti rotary file for tooth 22 (Protaper gold, Dentsply). Recapitulation was done using hand stainless steel K-files. The irrigation was done with 17% EDTA (Prime Dental) followed by 3% NaOCl (Prime Dental, India) using a 30-G side vented needle and intermittent flushing with 0.9% normal saline. Chlorhexidine 2% (Prime Dental) was used as a final irrigant during preparation, and 15% EDTA (Prime Dental) was used as a lubricant followed by ultrasonic activation of the irrigant (Ultra X, Orikam). Teeth were obturated with 4% gutta-percha cones using lateral condensation technique and MTA-based bio-ceramic sealer (Sealmax, Maarc Dental). Post space preparation was done using peeso reamer number two and heated hand pluggers whilst maintaining the apical plug dimensions of 5.5 mm for tooth 21 and five mm for tooth no. 22. Teeth were then temporized by placing PTFE tape in the canals and temporarily restored (Cavit, 3M ESPE).

Phase 2 (Surgical Procedure)

The following day, the patient reported no signs and symptoms. A surgical procedure was scheduled to reattach the crown fragments using a fiber post as an intra-radicular splint [10] to stabilize the fragments to the rest of the tooth structure. The patient was prescribed pre-operative medication (Amoxicillin/clavulanate potassium; Metronidazole; Diclofenac; Pantoprazole) one day prior to the surgical procedure. After local anesthesia administration with 2% lignocaine hydrochloride (1:200,000): adrenaline (bilateral infraorbital nerve block, nasopalatine nerve block), crevicular incisions (with blade no.12) were made from teeth 11 to 23 with vertical releasing incisions on either side of teeth (palatal aspect only). The mucoperiosteal flap was reflected followed by palatal osteotomy at the cervical portion of the teeth 21-22 (0.5-0.75 mm) to maintain the biological width. Intermittent betadine and saline irrigation were done to prevent dehydration of the flap (Figure 1F). Fiber post selection was done according to the last peeso reamer used (size #2). Vent holes were created in the crown fragments prior to the trial of the fiber post (to allow for the excess cement to be released) (Figure 1D3). The fragments were etched using 37% phosphoric acid gel (PRIME DENTAL) in total-etch and rinse mode. A universal bonding agent (3M ESPE Single Bond Universal Adhesive) was applied using applicator tips and cured using LED curing lamp (Woodpecker). The fiber post was etched with Hydrofluoric acid gel (Ultradent) and rinsed off followed by the application of a silane coupling agent (Ultradent). Dual cure resin cement (RelyX U200) was introduced into the canal, fiber post was cemented and cured using an LED curing lamp. The crown fragments were then cemented using flowable composite resin (3M ESPE Filtek Z350xt), tack cured and excess cement inter-proximally was removed followed by total light curing for 15 seconds. The mucoperiosteal flaps were re-approximated and sutured with 3-0 vicryl (Figures 1G, 1H).

Masking of the Interface Margins

Longitudinal grooves of 0.5mm depth were made at the tooth-fragment interface using tapered bur (TR-13EF, DIA). The surface area was etched, a bonding agent was applied, and light-curing was done for 20 seconds. Restorative composite (3M ESPE Z350xt) was added incrementally to camouflage the tooth-fragment interface. This step was followed by the finishing and polishing procedure using the graded abrasive discs and strips provided in the Shofu kit and polishing paste (Platina Hi-Gloss). A Ribbond splint was then given on the palatal aspect extending from teeth 11-23 to achieve stability and encourage periodontal healing (Figure 1I). Anterior occlusion was checked, and the interferences present were relieved.

He was evaluated for clinical and radiographic findings at the follow-up visits (Table 1).

**Table 1:** Follow-up checklist PDL space: Periodontal ligament space; PARL: Periapical radiolucency

SRL NO.	CLINICAL EXAMINATION	RADIOGRAPHIC EXAMINATION
1	Surgical site healing	Root-post-fragment interface integrity
2	Tender on percussion	Lamina dura
3	Pain on palpation	PDL space
4	Spacing at tooth-fragment interface	Signs of root resorption
5	Tooth mobility	PARL
6	Discoloration at tooth-fragment interface	
7	Probing depths	
8	Swelling	
9	Sinus tract	
10	Gingival papillary health	
11	Marginal gingiva	

**Figure 2:**
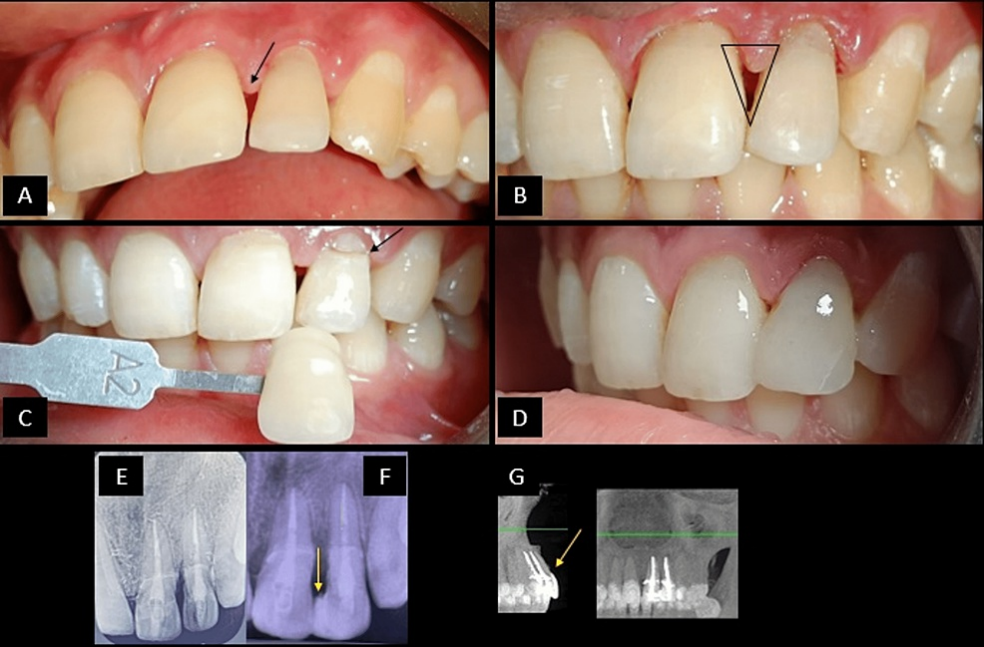
Follow-up clinical and radiographic images (ninth-month follow-up clinical images; 18th-month follow-up radiographic images). (A) Ninth-month clinical follow-up picture from labial view showing gingival papillary recession. (B) Gingival papillary recession between 21 and 22 has led to the formation of a gingival black triangle that is marked as seen in the image. (C) The shade selection is done using the VitaTM shade guide. The shade selected was A2. (D) Aesthetic composite splinting was done with composite restorative resin with respect to the inciso-proximal region of 21-22. (E) Three-month follow-up RVG showing intact tooth-fragment interface and absence of voids in the root canal suggesting the absence of any chance of microleakage development. (F) Nine-month follow-up RVG. (G) 18-month follow-up CBCT from sagittal and coronal view showing the maintenance of the intact tooth-fragment interface and absence of any periapical lesions.

At each follow-up visit, the checklist above was used to evaluate the overall condition of the teeth treated. It was noted that the overall status of teeth 21-22 is “good” and the patient is able to use the teeth well and is in a symptom-free state. However, at the ninth-month follow-up visit, there was gingival papillary recession noted w.r.t 21-22 region and composite were seen chipped off at the tooth-fragment interface (Figures 2A, 2B). Hence, an aesthetic composite splint in the 21-22 region was decided to be bonded along with laser crown lengthening on 22 so as to prevent the composite impingement on marginal gingiva. Composite shade selection was done using Vita shade guide (Figures 2C, 2D, 2F). The patient was then recalled for follow-up after 24 hours, one week, three months (Figure 2E), nine months and 18 months. At the 18th-month follow-up, the patient was clinically examined and a CBCT scan was advised. It was noted that there were no regressive changes seen radiographically. Clinically, it was noted that the gingival and periodontal health was found to be satisfactory with a stable tooth-fragment interface of both teeth with no mobility. Mild surface stains were noted on teeth and composite polishing was re-done (Figure 2G).

**Figure 3:**
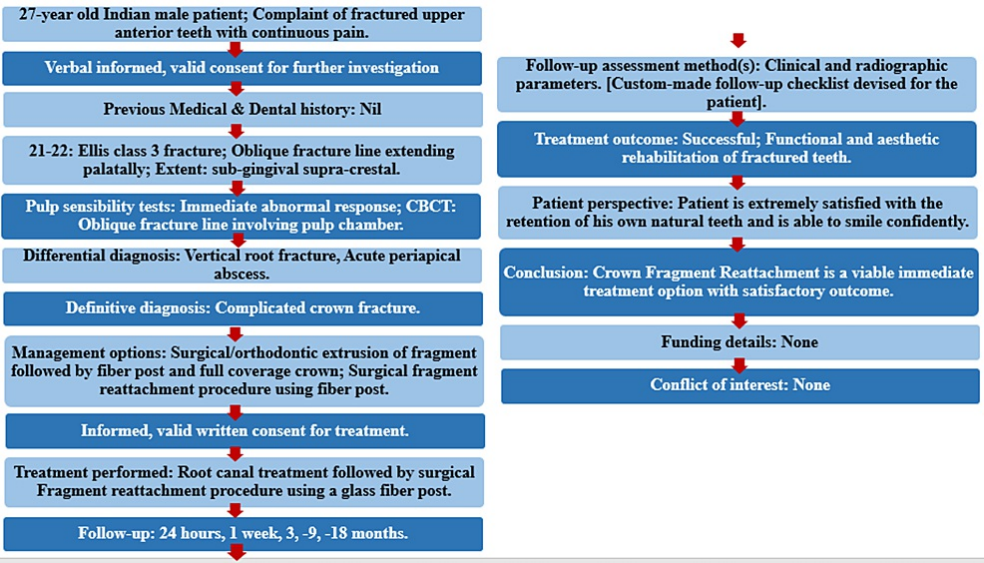
The PRICE 2020 flowchart showing the steps involved in the case report. For further details visit: http://pride-endodonticguidelines.org/price/ accessed on April 22, 2021. CBCT: Cone beam computed tomography

The PRICE 2020 flowchart shows the steps involved in the case report (Figure 3) [6].

## Discussion

Oblique coronal fractures involve the pulp chamber and extend sub-gingivally and sub- or supra-crestally, which may or may not have associated bone fractures. The treatment plan differs for each type of fracture. In this case, the teeth had oblique coronal fractures extending sub-gingivally and supra-crestally which necessitated the raising of the periodontal flap for the reattachment of the fragments for sufficient access and to avoid violation of biological width [11]. It has been shown in reports published earlier that reattachment of the fractured fragment is a valid treatment [12]. Reattachment with the original tooth structure results in the restoration of coronal and surface morphology in a material that wears at a similar rate as adjacent teeth. Chairside time for completion of the restoration is less as compared with the time needed for completion of a provisional or temporary restoration [2]. Reattachment of the fragment is superior as it provides better aesthetics with a life-like translucency, incisal edges abrade at a rate similar to that of the adjacent teeth, replacement of fractured portion in minimal time, a positive psychological response from the patient and it is an economical procedure [4]. A post and core procedure is chosen as it improves retention, stress distribution, and resistance to any further root fracture. The post acts as an intra-radicular splint [13], interlocks, and joins fragments and the rest of the tooth structure [14]. The translucent fiber post being luted with resin cement will enhance retention and makes provision for aesthetics along with the monobloc effect [15]. In teeth affected with oblique/transverse fractures, it is essential to stabilize them by splinting for a period of two to four weeks [16]. It helps in rapid and favorable healing patterns of the fractured segments and the periodontal tissues [17]. In the current case, fractured anterior teeth were splinted with “Ribbond,” a semi-rigid reinforced ribbon splint. In a recent case report published by Jaiswal et al. in 2022, the authors employed the use of fiber posts and the fractured crown fragment as it was in close approximation with the tooth structure. It has been noted in the literature that this is a viable treatment option for such complicated crown fractures where vent holes are created in the crown fragment which will lead to better adaptability of the fragment and post to the adhesive resin cement [18]. The highlight of this current case report is that the post-operative gingival recession was successfully managed using a diode laser and additive dentistry using composite restorative resin for the maintenance of white and pink esthetics.

## Conclusions

Adhesive dentistry has revolutionized modern clinical restorative dentistry. Thorough clinical and three-dimensional radiological examination of teeth is one of the major factors in understanding the extent of the fracture line in all the dimensions and hence, the treatment plan can be devised with utmost precision. The outcome of the treatment can be predicted accurately if the right imaging techniques have been used pre-operatively.

Complicated teeth fractures can be effectively managed by an interdisciplinary approach giving the best functional and aesthetic outcome. In the current case, successful management and maintenance of white and pink aesthetics have been portrayed. Hence, it can be concluded that the fractured crown fragments of teeth can be effectively utilized for the management of complicated fractures in conjunction with a suitable glass fiber post.
